# Supplementary material for: Exploring cell-specific miRNA regulation with single-cell miRNA-mRNA co-sequencing data
Source: BMC Bioinformatics. 2021 Dec 2;22:578. doi: 10.1186/s12859-021-04498-6 (PMC8641245; doi:10.1186/s12859-021-04498-6)
Supplement: Supplementary file 1 — Additional file 1. Figure S1 and Tables S1 and S2. [file 12859_2021_4498_MOESM1_ESM.docx]

**Exploring cell-specific miRNA regulation with single-cell miRNA-mRNA co-sequencing data**

Junpeng Zhang^1,2,*^, Lin Liu^3^, Taosheng Xu^4^, Wu Zhang^5^, Chunwen Zhao^2^, Sijing Li^2^, Jiuyong Li^3^, Nini Rao^1,*^, Thuc Duy Le^3,*^

^1^Center for Informational Biology, School of Life Science and Technology, University of Electronic Science and Technology of China, China, ^2^School of Engineering, Dali University, China, ^3^UniSA STEM, University of South Australia, Australia, ^4^Institute of Intelligent Machines, Hefei Institutes of Physical Science, Chinese Academy of Sciences, China, ^5^School of Agriculture and Biological Sciences, Dali University, China

*To whom correspondence should be addressed to Junpeng Zhang. Email: zhangjunpeng_411@yahoo.com

Correspondence may also be addressed to Nini Rao. Email: raonn@uestc.edu.cn

Correspondence may also be addressed to Thuc Duy Le. Email: thuc.le@unisa.edu.au

**Fig. S1** Cell-specific miRNA-mRNA interactions and hub miRNAs. (A) Number of predicted miRNA-mRNA interactions. (B) Percentage of validated miRNA-mRNA interactions. (C) Percentage of CML-related miRNA-mRNA interactions. (D) Percentage of CML-related hub miRNAs.

**Table S1.** Enrichment analysis of the rewired miRNA-mRNA modules associated with miR-17/92 family.

| ID | #GO | #KEGG | #Reactome | #Hallmark | #Cell marker | Enriched in CML or not? |
| --- | --- | --- | --- | --- | --- | --- |
| 1 | 0 | 0 | 0 | 0 | 0 | No |
| 2 | 7 | 0 | 8 | 1 | 0 | No |
| 3 | 31 | 1 | 9 | 1 | 0 | No |
| 4 | 52 | 54 | 28 | 2 | 8 | No |
| 5 | 0 | 16 | 3 | 0 | 0 | No |

**Table S2.** Hub cells in each cell-cell crosstalk network. The cell-cell crosstalk networks are generated in terms of network similarity and hub miRNA similarity.

| Terms | Hub cells | Degree |
| --- | --- | --- |
| Network similarity | K562_HalfCell_10 | 15 |
|  | K562_HalfCell_12 | 14 |
|  | K562_HalfCell_05 | 13 |
|  | K562_HalfCell_11 | 13 |
| Hub miRNA similarity | K562_HalfCell_15 | 11 |
|  | K562_HalfCell_02 | 8 |
|  | K562_HalfCell_13 | 8 |

**Table S3.** The identified cell-cell crosstalk modules. The cell-cell crosstalk networks are generated in terms of network similarity and hub miRNA similarity.

| Terms | Module ID | K562 HalfCell ID |
| --- | --- | --- |
| Network similarity | 1 | 01, 02, 03, 05, 07, 08, 10, 11, 12, 13, 14, 15, 16, 17, 18, 19, 20 |
| Hub miRNA similarity | 1 | 01, 02, 03, 05, 07, 08, 11, 12, 13, 14, 15, 16, 18, 19 |
